# Short-term *Aronia melanocarpa* extract supplementation improves cognitive performance: a randomized, double-blind, placebo-controlled cross-over study in healthy young adults

**DOI:** 10.1007/s00394-024-03381-3

**Published:** 2024-04-24

**Authors:** Sanne Ahles, Peter J. Joris, Jogchum Plat

**Affiliations:** 1 Department of Nutrition and Movement Sciences, Institute of Nutrition and Translational Research in Metabolism (NUTRIM), Maastricht University, 6200 MD Maastricht, The Netherlands; 2 BioActor BV, Gaetano Martinolaan 50, 6229 GS Maastricht, The Netherlands

**Keywords:** Cognition, Vascular function, Aronia, Anthocyanins, Human, Intervention

## Abstract

**Purpose:**

Evidence on the potential beneficial effects of anthocyanin-rich foods and supplements on cognitive performance is mainly based on acute or long-term studies in older adults. However, short-term studies focusing on a younger population are lacking. Therefore, short-term effects of *Aronia melanocarpa* extract (AME) supplementation on cognitive performance were investigated in healthy young adults. Potential underlying mechanisms were also addressed.

**Methods:**

A randomized, double-blind, placebo-controlled cross-over study was performed involving 35 apparently healthy young adults. Participants consumed AME (180 mg anthocyanins/day) or a placebo for 1 week, separated by at least 2 weeks of wash-out. Cognitive performance was assessed using the Cambridge Neuropsychological Test Automated Battery (CANTAB). Furthermore, arterial stiffness (carotid-to-femoral pulse wave velocity), retinal microvascular calibers (fundus photography), and serum brain-derived neurotrophic factor (BDNF) concentrations were measured at baseline and after 1 week.

**Results:**

Participants had a mean age of 25 ± 4 years and an average BMI of 23.4 ± 2.7 kg/m^2^. Compliance was excellent and the study product was well-tolerated. As compared to placebo, movement time was significantly reduced by 4.8% within the five-choice reaction time test after 1 week of AME supplementation (intervention effect: – 12 ms; p < 0.05). Memory and executive function did however not change. Serum BDNF concentrations were significantly higher after AME supplementation as compared to placebo (+ 5.7%; intervention effect: 1.8 ng/mL; p < 0.05). However, arterial stiffness and retinal microvascular calibers were not affected.

**Conclusion:**

Short-term AME supplementation beneficially affected cognitive performance as attention and psychomotor speed improved. Serum BDNF concentrations were increased, but vascular function markers were not affected.

**Clinical trial registration:**

The study was registered on Clinical Trials under NCT03793777 on January 4th, 2019.

**Supplementary Information:**

The online version contains supplementary material available at 10.1007/s00394-024-03381-3.

## Introduction

Due to population growth and ageing, the number of individuals dealing with age-related disorders such as cognitive impairment is increasing rapidly [1]. Cognitive capacity is the development of skills involved in learning, processing, and thinking. These skills generally peak in young adulthood, after which a gradual and normal decline occurs during the process of ageing. Consequently, during the lifespan, different cognitive goals are of interest. Specifically, it is key to increase the peak of their performance for young adults, while a prolonged peak and a slower decline are more relevant to maintain a healthy cognitive capacity later in life [2].

An important strategy to improve cognitive performance at all stages during the lifespan is through consuming healthy plant-based foods such as berries, which amongst others, are rich in anthocyanins. These flavonoids are recognized for their antioxidant capacity, and their potential role in the prevention of various chronic diseases such as cardiovascular disease, diabetes, and obesity [3]. Protective effects on characteristics of metabolic syndrome and glycemic control have already been reported in detail [4, 5]. We have previously reported beneficial effects of long-term (i.e., 24 weeks) supplementation with anthocyanin-rich chokeberry extract (*Aronia melanocarpa* extract (AME)), on cognitive performance in healthy middle-aged individuals [6]. Moreover, we recently systematically reviewed effects of berry anthocyanin interventions on cognitive performance [7]. It was concluded that memory was improved mostly in older adults, while evidence for an improvement in the attention and psychomotor speed domain was strongest in younger adults. Likewise, enhanced executive function was observed in most studies with children but was less apparent in studies involving (older) adults. However, these conclusions were mainly based on results from acute (8 studies; 1.25–8 h) or long-term (9 studies; 4–24 weeks) interventions. Only one short-term study using a New Zealand Blackcurrant intervention for 1 week in older adults was included but did not observe any significant effects on cognitive performance [8]. Consequently, evidence from short-term studies is still lacking.

Diet-induced changes in vascular function and brain-derived neurotrophic factor (BDNF) may play an important role in the development of cognitive benefits [9, 10]. BDNF increases synaptic plasticity and neurogenesis, which are involved in the processes of learning and memory [9]. Furthermore, a prior study in ageing rats has observed that anthocyanin-induced improvements in spatial working memory and psychomotor performance may be due to the regulation of proteins related to synaptic plasticity, such as BDNF [11]. In our systematic review, we also observed beneficial effects of berry anthocyanins for various markers of vascular function [7]. These markers are thought to be related to cognitive performance through mechanisms such as arterial stiffness and blood flow [12, 13]. However, well-controlled human studies assessing both cognitive performance and potential underlying mechanisms, such as improvements in serum BDNF concentrations or vascular characteristics (e.g., arterial stiffness and retinal microvascular calibers), are scarce.

The aim of this randomized, double-blind, placebo-controlled, cross-over study was to determine short-term (1 week) effects of AME supplementation on the main cognitive domains (attention and psychomotor speed, memory, and executive function) in healthy young adults. Moreover, we assessed effects of AME supplementation on potential underlying mechanisms.

## Materials and methods

### Study population

Apparently healthy young adults (aged 18–35 years) with a body mass index (BMI) between 18.5 and 30.0 kg/m^2^ were recruited through local advertisements and social media. Participants were invited for a screening visit to evaluate their eligibility and to ensure proper familiarization with the cognitive performance measurements. Exclusion criteria were pre-existing neurological illnesses, use of medication or dietary supplements that might influence endpoints of the study (such as antidepressants or blood pressure medication), high blood pressure (> 140 mmHg systolic and/or > 90 mmHg diastolic blood pressure), pregnancy, smoking, and abuse of alcohol (> 20 alcoholic units/week) or drugs. All participants gave written informed consent before data collection. The study was approved by the Medical Ethics Committee of University Hospital Maastricht and Maastricht University (METC azM / UM) and performed at the University of Maastricht between October 2020 and April 2021 in accordance with the 1964 Declaration of Helsinki and its later amendments. The study was registered online at ClinicalTrials.gov as NCT03793777.

### Study design

A randomized, double-blind, placebo-controlled, cross-over study was performed. After successful screening, each participant was randomly allocated to receive 1 week of either AME or placebo, separated by a wash-out period of at least 2 weeks. At baseline and after 1 week of intervention, participants visited the research facilities. Anthropometric parameters, cognitive performance, and vascular function measurements were performed in temperature-controlled rooms of the Metabolic Research Unit Maastricht (MRUM), and fasted blood samples were collected. All study visits were performed in the morning in a fasted state, and different study visits for each participant occurred at the same time of the day. An electronic data capture system (Castor EDC, Amsterdam, the Netherlands) was used for data collection and the study was monitored by the Clinical Trial Center Maastricht (CTCM). The randomization schedule was computer-generated and was performed by an independent researcher, using random and concealed block sizes, and stratifying for sex.

During the study, participants were provided with a list of foods containing anthocyanins and were instructed to refrain from these products during the study period. Furthermore, participants were instructed to avoid strenuous physical activity and alcohol on the 2 days preceding each test day, and to arrive fasted in the morning of the measurements.

### Intervention

The study product was an AME containing 24% anthocyanins (14.4% cyanidin-3-galactoside and 9.6% of other cyanidin-3-glycosides, [i.e. cyanidin-3-arabinoside, cyanidin-3-xyloside, cyanidin-3-glucoside]) which was provided by BioActor BV (Brainberry^®^; Maastricht, The Netherlands). A daily dose of 750 mg AME was provided in three capsules containing in total 180 mg anthocyanins per day. This dose was based on previous acute and short-term research with anthocyanin interventions in young populations showing improvements in cognitive performance [14–16]. Identical numbers of cellulose-containing capsules were used as placebo. AME and placebo capsules were opaque and uniform in appearance. The capsule jars each contained 30 capsules and were blinded, displaying only the participant number and the intervention period on the label. Participants were instructed to consume three capsules daily before breakfast, with 200 mL water, and to note daily intake and any deviations in a supplementation logbook. Remaining capsules at the end of the intervention period were returned to the study facility to monitor compliance, which was considered valid if > 85%.

### Cognitive performance and mood measurements

Cognitive performance was assessed using the Cambridge neuropsychological test automated battery (CANTAB) [17]. This is a validated and computerized assessment that we have previously used to assess three main cognitive domains: attention and psychomotor speed, memory, and executive function [18]. The motor screening test (MOT) was performed as the first test in every session and was used to get familiar with the CANTAB system. Attention and psychomotor speed were assessed using the five-choice reaction time (RTI) test. In the RTI test, the time from stimulus to release of the response button (reaction time) and the time from release of the response button to selection of target (movement time) of correctly assessed trials is determined. Measurements in the memory domain were the delayed matching to sample (DMS) and paired associates learning (PAL) test. For the DMS, the combined percentage of correct trials after 0 s, 4 s, and 12 s of delay were recorded. The PAL included a first-attempt memory score and total errors on the 12-item trial. Executive function was measured with the multitasking test (MTT) and spatial working memory test (SWM). The MTT provided the mean response latency, incongruency cost, multitasking cost, and total errors. For the SWM, total errors on the 12-item trial and strategy score were included.

Mood was assessed using 100 mm visual analogue scales, including eight mood states: afraid, angry, confused, energetic, happy, sad, tense, and tired [19]. The cognitive failures (CFQ) was used to assess subjective cognitive failure in daily life [20].

### Blood pressure and vascular function measurements

Measurements were performed in a supine position after an acclimatization period of at least ten minutes. First, office blood pressure was determined in fourfold, of which the first measurement was disregarded (Omron 705IT, Hoofddorp, The Netherlands). Radial artery pulse wave analyses (PWA) of the brachial artery were performed in triplicate to determine mean arterial pressure (MAP), near the elbow and wrist of the arm using a tonometer (SphygmoCor v9, AtCor Medical, West Ryde, Australia). Consequently, the central augmentation index corrected for heart rate (cAIxHR75) was calculated using the difference between the first and second peaks of the central arterial waveform. Furthermore, carotid-to-femoral pulse wave velocity (_cf_PWV) was measured in triplicate as a measure of arterial stiffness, as described previously [21].

Microvascular retinal calibers were determined using a fundus camera (Topcon TRC-NW-300, TopCon Co., Tokyo, Japan), Vascular images of the optic disc were taken and images from both study periods were analyzed simultaneously using Interactive Vessel Analyzer software (IVAN, University of Wisconsin, Wisconsin, USA) to ensure that the selected segments were identical in all images of a participant. Using the Parr-Hubbard formula [22], the mean central retinal arteriolar and venular equivalents (CRAE and CRVE, respectively) and the arteriolar-to-venular ratio (AVR) were calculated.

### Biochemical analyses

Fasting blood samples were collected in serum separator tubes (BD Vacutainer, NJ, USA). Serum tubes were centrifuged after exactly one hour of coagulation (room temperature, 1300xg, 10 min) as clotting time could affect BDNF concentrations in serum [23]. Serum aliquots were stored for further analysis at the end of the trial. BDNF concentrations were determined using an enzyme-linked immunosorbent assay (Duoset ELISA, R&D systems, Minneapolis, MN, USA), according to the manufacturer’s protocol.

### Statistical analyses

An expected effect size of 0.602 was determined, based on the change from baseline on repetition seven of the digit vigilance test (a measure of cognitive function, which was defined as the primary study outcome) in a study by Watson et al. [24]. Using a power of 90%, and a two-sided alpha of 0.05, a sample size of at least 31 participants was required. To account for expected drop-outs, a total of 36 participants were included. Statistical analyses were performed using IBM SPSS Statistics (26.0, IBM Corporation, Armonk, NY, USA).

Statistical analyses were performed using linear mixed models including intervention, period, and sex as fixed factors, baseline values as covariate, and participant and intercept as random factors. Raw scores of the post-intervention measures were used with the corresponding baseline values as covariates. The three-way (intervention*period*sex) and two-way interactions (intervention*period, intervention*sex, and period*sex) were omitted from the model if not significant following a top-down approach. Pearson correlations were determined between significant changes observed in cognitive performance and those in serum BDNF concentrations. Data were reported as unadjusted means ± SDs, unless stated otherwise. For all analyses, two-sided p values < 0.05 were considered statistically significant.

## Results

In total, 39 participants were screened for eligibility, of which three were excluded due to medication that was not allowed (n = 2) or smoking (n = 1). Consequently, 36 participants were randomized to start either with the Aronia extract or placebo (Fig. S1). Two participants dropped out due to personal reasons. One participant before the first baseline measurement, and one participant during the placebo period. Collected data from 35 (14 men and 21 women) participants was used for all analyses. Study participants were 25 ± 4 years old and had an average BMI of 23.4 ± 2.7 kg/m^2^. Baseline characteristics are shown in Table 1. Compliance was considered excellent, as 34 out of 35 participants fully adhered to both allocated interventions. Removing one participant that consumed more capsules than instructed (143%) from the statistical analyses did however not affect the study outcomes. Therefore, we finally decided to also include that participant in all analyses. The study product was well-tolerated, and no serious adverse events or protocol deviations were reported.

**Table 1:** Baseline characteristics of study participants^a^

	Study participants (n = 35)
Men (n; %)	14; 40%
Age (years)	25 ± 4
BMI (kg/m^2^)	23.4 ± 2.7
Systolic blood pressure (mmHg)	106 ± 2
Diastolic blood pressure (mmHg)	68 ± 1
Heart rate (bpm)	62 ± 2

*BMI* body mass index

^a^Data is displayed as mean ± SDs or as a percentage

**Fig. 1:**
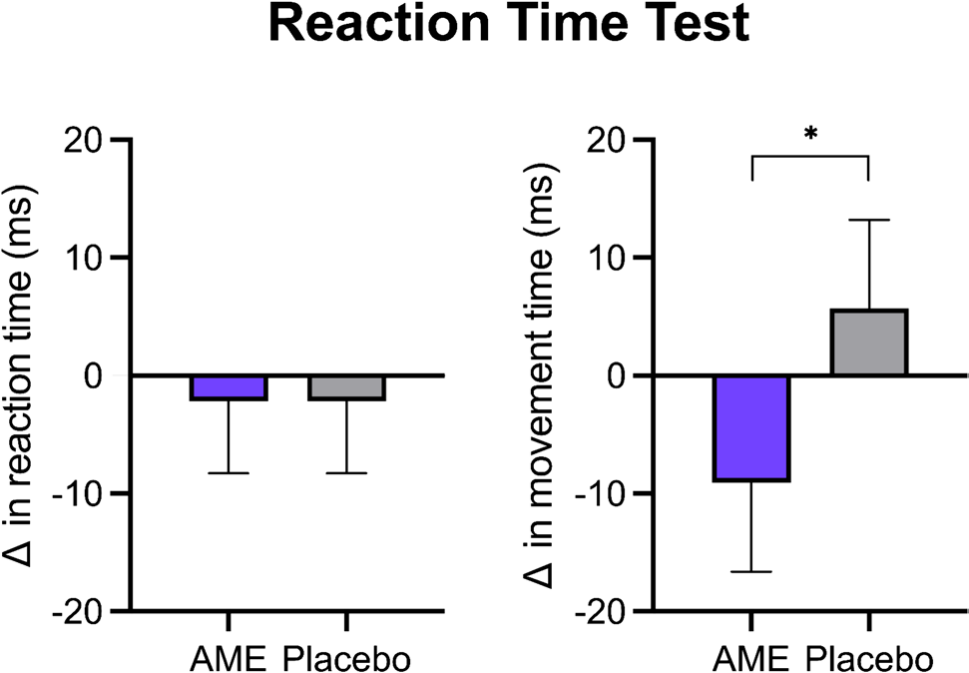
The change in reaction time and movement time during the five-choice reaction time test following 1 week of AME or placebo supplementation. Data are presented as mean change from baseline ± SEM. Analysis was performed with a linear mixed model using intervention, period, and sex as fixed factors, and baseline values as covariate. *Indicates p < 0.05

Results of the five-choice reaction time test, within the domain of attention and psychomotor speed, are displayed in Fig. 1. The reaction time, reflecting the time from the stimulus appearance to the release of the response button, was not significantly different between the AME and placebo period (– 5 ms [– 15, 4]; p = 0.253). However, the movement time, defined as the time from release of the response button to selection of the target, was significantly improved by 4.8% after 1 week of AME supplementation, as compared with placebo (intervention effect of – 12 ms [– 21, – 2]; p = 0.019).

Within the memory domain, a significant intervention*sex interaction for total errors on the PAL test (p = 0.019) was observed with less errors in women after AME supplementation as compared to men. No significant effects of AME supplementation were observed for the DMS and PAL tests. Similarly, the MTT and SWM tests, reflecting performance within the executive function domain were not significantly affected by AME supplementation (see Table 2). However, for the MTT incongruency cost, a significant intervention*sex interaction was observed (p = 0.028), indicating an improved score for men after AME supplementation as compared to women. Mood and subjective cognition were not changed after AME supplementation (Table S1).

**Table 2:** Cognitive performance following 1 week of AME or placebo supplementation^a^

Cognitive tests	AME		Placebo		Intervention effect^b^
	Baseline	After 1 week	Baseline	After 1 week	
Attention and Psychomotor Speed					
RTI—reaction time (ms)	378 ± 34	374 ± 37	378 ± 38	378 ± 39	– 5 [– 15, 4]; p = 0.253
RTI—movement time (ms)	248 ± 43	239 ± 46	237 ± 42	242 ± 46	– 12 [ – 21, – 2]; p = 0.019*
Memory					
DMS—total correct (%)	93 ± 7	92 ± 8	90 ± 10	91 ± 9	0 [– 3, 3]; p = 0.879
PAL – first-attempt memory score	17 ± 3	18 ± 2	17 ± 1	18 ± 2	0 [– 1, 0]; p = 0.299
PAL—total errors 12 trial	7 ± 9	7 ± 9	6 ± 6	5 ± 7	N/A^d^
Executive Function					
MTT—incongruency cost (ms)	57 ± 38	47 ± 37	58 ± 45	44 ± 39	N/A^d^
MTT—median latency (ms)	533 ± 78	517 ± 74	542 ± 78	514 ± 65	12 [– 3, 28]; p = 0.113
MTT—multitasking cost^c^	85 ± 78	59 ± 40	89 ± 74	69 ± 62	– 8 [– 29, 14]; p = 0.478
MTT—total errors	2 ± 2	3 ± 3	3 ± 3	2 ± 2	1 [0, 2]; p = 0.151
SWM—total errors 12 trial	18 ± 14	17 ± 13	19 ± 14	16 ± 13	2 [– 3, 7]; p = 0.361
SWM—strategy score^c^	8 ± 4	8 ± 5	8 ± 5	9 ± 5	0 [– 2, 1]; p = 0.556

*DMS* delayed matching to samples test, *MTT* multitasking test, *PAL* paired associates learning test, *RTI* reaction time test, *SWM* spatial working memory test

*Indicates p < 0.05

^a^Data are presented as means ± SDs

^b^Analysis was performed with a linear mixed model using intervention, period, and sex as fixed factors, and baseline values as covariate

^c^Low scores indicate better performance

^d^Treatment effects were not analyzed as a significant intervention*sex interaction was observed.

**Fig. 2:**
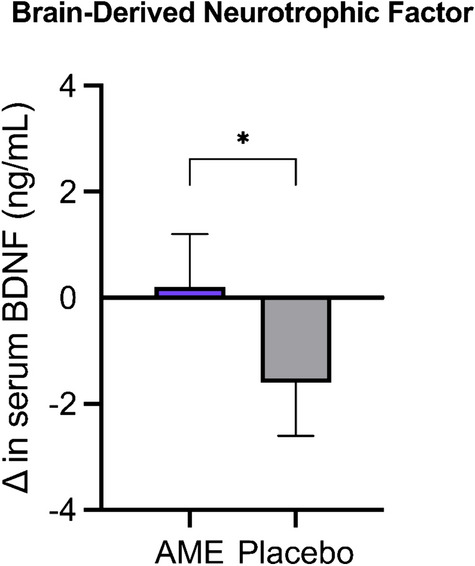
The change in serum brain-derived neurotrophic factor (BDNF) concentrations following 1 week of AME or placebo supplementation. Data are presented as mean change from baseline ± SEM. Analysis was performed with a linear mixed model using intervention, period, and sex as fixed factors, and baseline values as covariate. *Indicates p < 0.05. *BDNF* brain-derived neurotrophic factor

Serum BDNF concentrations were significantly higher after AME supplementation, as compared with placebo (+ 5.7%; intervention effect of 1.8 ng/mL [0.4, 3.3]; p = 0.013) (Fig. 2**, **Table S2). Observed changes in serum BDNF concentrations did not correlate with changes in movement time on the RTI (r = – 0.061, p = 0.623).

In Table 3, effects of AME supplementation on office blood pressure and vascular function measurements are shown. No significant effects on office systolic and diastolic blood pressure levels, heart rate, or mean arterial pressure were observed. Also, arterial stiffness, assessed with _cf_PWV and cAIxHR75 did not change in the AME versus placebo period. In addition, no changes in the retinal microvascular calibers (i.e., CRAE/CRVE/AVR) were observed after 1 week of supplementation with AME as compared to placebo (all p > 0.05).

**Table 3:** Blood pressure and vascular function measurements following 1 week of AME or placebo supplementation^a^

Vascular function	AME		Placebo		Intervention effect^b^
	Baseline	After 1 week	Baseline	After 1 week	
Blood Pressure					
SBP (mmHg)	106 ± 11	105 ± 10	107 ± 10	106 ± 10	0 [– 3, 3]; p = 0.999
DBP (mmHg)	67 ± 6	67 ± 6	68 ± 7	67 ± 7	0 [– 2, 2]; p = 0.753
Heart rate (bpm)	62 ± 9	63 ± 9	62 ± 9	62 ± 11	1 [– 1.8, 3.3]; p = 0.552
MAP (mmHg)	81 ± 8	81 ± 8	82 ± 7	81 ± 8	0 [– 2, 2]; p = 0.881
Arterial Stiffness					
cAIxHR75 (%)	– 1.3 ± 10.7	– 3.0 ± 8.8	– 2.4 ± 15	– 2.3 ± 10	– 1.7 [– 4.3, 0.8]; p = 0.173
_cf_PWV (m/s)	7.5 ± 1.0	7.5 ± 1.0	7.6 ± 1.1	7.4 ± 0.9	0.1 [– 0.3, 0.4]; p = 0.768
Retinal Microvascular Calibers					
CRAE (μM)	125 ± 21	125 ± 21	124 ± 21	123 ± 22	0 [– 2, 2]; p = 0.758
CRVE (μM)	218 ± 15	218 ± 14	219 ± 14	218 ± 14	0 [– 2, 2]; p = 0.957
AVR	0.57 ± 0.09	0.57 ± 0.10	0.57 ± 0.09	0.57 ± 0.10	0.00 [– 0.01, 0.01]; p = 0.746

*AVR* arteriolar-to-venular-ratio, *cAIxHR75* central augmentation index adjusted for heart rate, *CRAE* central retinal arteriolar equivalent, *CRVE* central retinal venular equivalent, *DBP* diastolic blood pressure, *MAP* mean arterial pressure, _*cf*_*PWV* carotid-to-femoral pulse wave velocity, *SBP* systolic blood pressure

^a^Data are presented as means ± SDs

^b^Analysis was performed with a linear mixed model using intervention, period, and sex as fixed factors, and baseline values as covariate

## Discussion

In this randomized, double-blind, placebo-controlled, cross-over trial involving healthy young adults, the primary aim was to assess a potential change in cognitive performance following AME versus placebo supplementation. In addition, we explored potential underlying mechanisms. It was shown that 1 week of AME supplementation improved attention and psychomotor speed, compared to placebo whereas other cognitive domains remained unchanged. Serum BDNF concentrations were significantly increased as compared to placebo. However, these changes in BDNF levels were not correlated to the significant improvements observed in movement time. Vascular function was not affected.

Previously, a systematic review on dietary supplements and cognitive performance in healthy young adults showed that flavonoid supplementation resulted in increased attention, but not in memory or executive function [25]. Woods et al. [26] performed a study involving 1466 participants between the age of 18–65 years old, where it was shown that choice reaction time latency, assessed with a digital test battery, decreases with age by approximately 3 ms per year. In the current study, we observed a 12 ms improvement in movement time on the five-choice reaction test, which reflects improved psychomotor speed, which may contribute to delaying this age-related cognitive decline. Based on the outcomes of our systematic review assessing effects of berry anthocyanins, we have previously already provided evidence for an improved attention and psychomotor speed in young adults [7]. However, improvements in other cognitive domains, such as memory, seem unlikely within this specific age group. A similar pattern was observed in our previous long-term AME intervention study which was performed with healthy middle-aged adults for 24 weeks [6]. Here, psychomotor speed, assessed with the grooved pegboard test, was significantly improved in the AME intervention as compared to placebo. Thus, young adults and middle-aged adults appear to be sensitive to changes in attention and psychomotor speed. A study by Hartshorne and colleagues [27] combined data from almost 50,000 participants to determine when cognitive capacity is at the highest level. They concluded that even though cognitive capacity is at its highest in young adulthood, a difference between performance in cognitive domains can still be observed. Cognitive abilities, such as memory, are thought to peak in young adulthood while measures for attention peak later in life. This suggests that there is still room for improvements in attention and psychomotor speed in younger adults, while older adults may benefit more from improvements in memory.

Alternatively, 1 week of supplementation may not have been long enough to induce changes in memory or executive function, which would suggest that the observed improvement in the domain of attention and psychomotor speed is regulated through different mechanisms as compared to potential changes observed in memory or executive function domains. Current evidence proposes that flavonoids can indeed influence various processes that could translate into an improved cognitive performance. For example, in short-term and longer-term studies, increases in regional cerebral blood flow – a marker of brain vascular function are considered to be an important mechanism underlying improvements observed in cognitive performance [28]. Here, various brain regions related to different cognitive domains could be affected, depending on factors such as the included study population and duration, or the intervention. Moreover, it has been shown that flavonoids beneficially affect vascular function and elevate BDNF concentrations [29]. Nevertheless, it is currently not known if and how these mechanisms can differentially affect specific cognitive abilities.

An important question is how the observed improvement in movement time could be explained. In line with the observations from longer-term flavonoid interventions in general [23], we here show that serum BDNF concentrations were higher after AME supplementation versus placebo. However, improvements in serum BDNF concentrations did not correlate with the observed beneficial effects on cognitive performance. Therefore, a clear mechanistic link between BDNF concentrations and cognition could not be established. Since our study was not sufficiently powered for thorough correlation analyses, this outcome should however be interpreted with caution. Previously, we did not observe significant changes in serum BDNF concentrations [6], which may be due to differences in intervention products, study duration (24 weeks vs 1 week), or study population (40–60 years old vs 18–35 years old). So far, available data investigating anthocyanins and BDNF-related pathways as an underlying mechanism for cognitive performance are limited to cell and animal studies [30–32]. Inconsistent with our results, these studies reported a positive correlation between anthocyanin-induced changes in BDNF with changes in cognition. BDNF concentrations were however measured in the brain, and (diet-induced changes in) BDNF concentrations may be different in the brain and the periphery [23]. Alternatively, anthocyanins could have direct effects in the brain since they can cross the blood–brain barrier from the circulation into the brain. Here, they could theoretically affect neurogenesis and synaptic plasticity [33]. This hypothesis needs however further study.

Flavonoids are also known for their vasoprotective effects, which could potentially also explain the observed cognitive improvements. Through an improved vascular function, anthocyanins may contribute to better nutrient and oxygen supply to the brain, which in turn could support optimal cognitive function. However, we observed no effects on arterial stiffness and retinal microvascular calibers. Various studies did show short-term effects of anthocyanin supplementation on arterial stiffness. In a study performed in older adults, _cf_PWV decreased after 7 days of blackcurrant extract intake [34]. Moreover, 2 weeks of supplementation with purple potato anthocyanins also resulted in a decreased _cf_PWV in healthy adults [35]. Importantly, observed improvements in PWV are most likely caused by decreased blood pressure levels, which did not change in the current study, and they reflect functional rather than structural vascular differences [36]. Retinal microvascular calibers were also assessed as there is evidence suggesting a mechanistic link with changes in cognitive performance [37, 38]. However, no significant changes were observed after AME supplementation. To the best of our knowledge, the effects of flavonoid supplementation on retinal microvascular caliber profiles have not been evaluated before. Previous research with anthocyanins has mainly focused on diabetic retinopathy or retinal damage [39]. The lack of an effect in our study may relate to the young population included, as they typically do not show any signs of microvascular dysfunction yet. Nevertheless, further studies are needed to verify this in different study populations (e.g. older healthy individuals, or individuals with (micro)vascular complications).

A strength of this study was the focus on cognitive performance as assessed using the validated, digital, standardized CANTAB system, which is considered a sensitive method to detect cognitive performance changes after dietary interventions [40]. Furthermore, we investigated different potential mechanisms of action. This approach contributed to a more comprehensive understanding of health effects related to anthocyanin intake. We were however unable to identify a vascular mechanism involved in the improvement in cognitive performance. Therefore, the effects of anthocyanins on cognitive performance might be directly regulated locally through an improved brain vascular function, which was not measured in this study. Cerebrovascular measurements, such as cerebral blood flow, are associated with cognitive performance and could be of interest for future intervention studies [13]. Moreover, Pilipović et al. [41] proposed that a combination of various separate processes, such as an increased blood flow and protein synthesis, protection against oxidative stress and neuro-excitotoxicity, could induce neuroprotective effects. These mechanisms of action could also be considered in the future. Furthermore, individual variations in anthocyanin bioavailability and metabolism may be interesting to consider in future research [42]. It has often been reported that anthocyanin bioavailability is rather low and could be affected by genetic factors, diet, age, sex, microbiota composition, etc. Consequently, it is of interest to identify individual responses to anthocyanin intake, which is important to determine optimal dietary intakes for different subpopulations.

In conclusion, 1 week of AME supplementation improved cognitive performance, which was reflected by a shorter movement time on the five-choice reaction time test. Serum BDNF concentrations were also improved, but changes did not correlate with the improvements observed in the domain of attention and psychomotor speed. No changes were observed on vascular function. Therefore, we suggest that other vascular parameters such as markers of endothelial function or brain vascular function should be further investigated to explore potential underlying mechanisms into more detail.

### Supplementary Information

Below is the link to the electronic supplementary material.

Supplementary file1 (PDF 81 KB)

Supplementary file2 (DOCX 95 KB)

## Data Availability

The data in this current study are available from the corresponding author upon reasonable request.

## Abbreviations

AME: *Aronia melanocarpa* Extract
BDNF: Brain-derived neurotrophic factor
BMI: Body mass Index
CAIxHR75: Central augmentation index corrected for heart rate
CANTAB: Cambridge Neuropsychological Test Automated Battery
_cf_PWV: Carotid-to-femoral pulse wave velocity
CRAE: Central retinal arteriolar equivalent
CRVE: Central retinal venular equivalent
CTCM: Clinical Trial Center Maastricht
DMS: Delayed matching to sample test
MAP: Mean arterial pressure
MOT: Motor screening test
MTT: Multitasking test
MRUM: Metabolic Research Unit Maastricht
PAL: Paired associates learning test
PWA: Pulse wave analyses
RTI: Reaction time test
SWM: Spatial working memory test
