# Supplementary material for: Short-term Aronia melanocarpa extract supplementation improves cognitive performance: a randomized, double-blind, placebo-controlled cross-over study in healthy young adults
Source: Eur J Nutr. 2024 Apr 24;63(5):1545–53. doi: 10.1007/s00394-024-03381-3 (PMC11329521; doi:10.1007/s00394-024-03381-3)
Supplement: Supplementary file 2 — Supplementary file2 (DOCX 95 KB) [file 394_2024_3381_MOESM2_ESM.docx]

**Supplemental information**


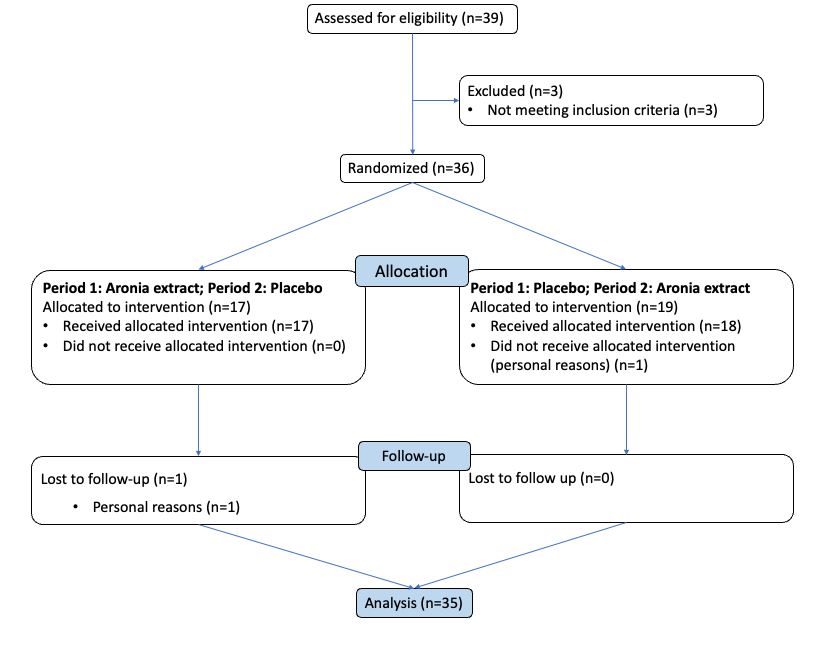


**Fig. S1** Consolidated Standards of Reporting Trials (CONSORT) flow diagram of the study.

**Table S1** Mood and subjective cognition following one week of AME or placebo supplementation^1^.

|  | **AME** | | **Placebo** | | **Intervention effect^2^** |
| --- | --- | --- | --- | --- | --- |
|  | **Baseline** | **After 1 week** | **Baseline** | **After 1 week** |  |
| **Mood** | | | | | |
| Afraid | 47.8 ± 7.9 | 47.5 ± 7.7 | 46.8 ± 3.9 | 47.3 ± 9.0 | -0.6 [-3.5, 2.4]; p=0.710 |
| Angry | 44.9 ± 4.7 | 43.8 ± 2.2 | 44.0 ± 3.0 | 45.2 ± 7.4 | -2.0 [-4.4, 0.4]; p=0.101 |
| Confused | 45.7 ± 5.0 | 45.8 ± 4.2 | 46.0 ± 4.9 | 46.3 ± 6.4 | -0.1 [-1.7, 1.5]; p=0.871 |
| Energetic | 45.0 ± 10.0 | 45.8 ± 9.7 | 44.2 ± 8.7 | 44.7 ± 10.9 | 0.2 [-3.1, 3.5]; p=0.884 |
| Happy | 43.1 ± 10.3 | 44.5 ± 10.6 | 43.9 ± 11.1 | 44.9 ± 10.7 | -0.2 [-3.0, 3.5]; p=0.891 |
| Sad | 45.5 ± 5.0 | 45.4 ± 4.1 | 45.9 ± 5.3 | 46.8 ± 8.7 | -1.4 [-4.4, 1.7]; p=0.368 |
| Tense | 47.7 ± 8.9 | 45.7 ± 7.5 | 46.3 ± 6.8 | 45.4 ± 8.9 | -0.5 [-3.6, 2.6]; p=0.741 |
| Tired | 43.8 ± 6.9 | 45.5 ± 10.6 | 45.7 ± 7.5 | 46.7 ± 8.1 | -0.2 [-2.8, 2.4]; p=0.869 |
| **Subjective Cognitive Failure** | | | | | |
| Score | 24 ± 11 | 19 ± 11 | 25 ± 14 | 20 ± 12 | 0 [-4, 3]; p=0.859 |

^1^ Data are presented as means ± SDs. ^2^ Analysis was performed with a linear mixed model using intervention, period, and sex as fixed factors, and baseline as covariate.

**Table S2** Serum brain-derived neurotrophic factor (BDNF) concentrations following one week of AME or placebo supplementation^1^.

|  | **AME** | | **Placebo** | | **Intervention effect^2^** |
| --- | --- | --- | --- | --- | --- |
|  | **Baseline** | **After 1 week** | **Baseline** | **After 1 week** |  |
| BDNF (ng/mL) | 31.8 ± 6.0 | 32.0 ± 6.0 | 33.0 ± 5.3 | 31.4 ± 5.9 | **1.8 [0.4, 3.3]; p=0.013*** |

^1^ Data are presented as means ± SDs. ^2^ Analysis was performed with a linear mixed model using intervention, period, and sex as fixed factors, and baseline as covariate. * indicates p < 0.05. Abbreviations: BDNF: brain-derived neurotrophic factor.
